# m ^6^A methylation in cellular senescence of age-associated diseases

**DOI:** 10.3724/abbs.2023107

**Published:** 2023-07-03

**Authors:** Pan Gao, Feng Yao, Jin Pang, Kai Yin, Xiao Zhu

**Affiliations:** 1 Guangxi Key Laboratory of Diabetic Systems Medicine Guilin Medical University Guilin 541100 China; 2 The Fifth Affiliated Hospital of Southern Medical University Guangzhou 510900 China

**Keywords:** cellular senescence, N ^6^-methyladenosine, age-associated diseases

## Abstract

Cellular senescence is a state of irreversible cellular growth arrest that occurs in response to various stresses. In addition to exiting the cell cycle, senescent cells undergo many phenotypic alterations, including metabolic reprogramming, chromatin rearrangement, and senescence-associated secretory phenotype (SASP) development. Furthermore, senescent cells can affect most physiological and pathological processes, such as physiological development; tissue homeostasis; tumour regression; and age-associated disease progression, including diabetes, atherosclerosis, Alzheimer’s disease, and hypertension. Although corresponding anti-senescence therapies are actively being explored for the treatment of age-associated diseases, the specific regulatory mechanisms of senescence remain unclear. N ^6^-methyladenosine (m ^6^A), a chemical modification commonly distributed in eukaryotic RNA, plays an important role in biological processes such as translation, shearing, and RNA transcription. Numerous studies have shown that m ^6^A plays an important regulatory role in cellular senescence and aging-related disease. In this review, we systematically summarize the role of m ^6^A modifications in cellular senescence with regard to oxidative stress, DNA damage, telomere alterations, and SASP development. Additionally, diabetes, atherosclerosis, and Alzheimer’s disease regulation via m ^6^A-mediated cellular senescence is discussed. We further discuss the challenges and prospects of m ^6^A in cellular senescence and age-associated diseases with the aim of providing rational strategies for the treatment of these age-associated diseases.

## Introduction

With the continuous development of medical technology, the average life span has been significantly extended [1], and the world’s aging population is increasing [2]. Epidemiological studies indicate that approximately 11% of the world’s population is over 60 years of age, and this proportion is estimated to exceed 20% by the middle of the 21st century. This increase in the aging population has been associated with a concomitant increase in the incidence of age-associated diseases such as diabetes, atherosclerosis (AS), Alzheimer’s disease (AD), and hypertension [3]. A typical feature of aging in biological individuals is the continuous accumulation of senescent cells in the body [ 4, 5] . Cellular senescence is a state of irreversible cellular growth arrest that is accompanied by mitosis termination, cell cycle arrest, and proliferation marker reduction [ 4, 6] . There are many causes of cellular senescence, including oxidative stress, DNA damage, telomere shortening, altered telomerase activity and structure, and oncogenic stress response onset [ 5, 7] . At an organismic level, cellular senescence has advantages and disadvantages. Nonetheless, cellular senescence facilitates embryonic development, tissue repair and regeneration and promotes cellular reprogramming [3]. However, cellular senescence can aggravate oxidative stress, proinflammatory factor expression, mitochondrial damage, and DNA damage; ultimately, all of these processes can further affect the normal function of organs and tissues, causing the development of age-related diseases. Although many prevention and treatment options for cellular senescence and age-associated diseases have been explored, the specific regulatory mechanisms of these treatments remain unclear.

N ^6^-methyladenosine (m ^6^A) is a common chemical modification distributed throughout eukaryotic RNAs. N6-methyladenosine and its associated enzymes (FTO, ALKBH5, METTL3, METTLI14, WTAP, and YTHDF2) play important roles in biological processes, such as translation, shearing, and RNA transcription [8]. With advances in epigenomics and sequencing technologies, the exact role of m ^6^A in cellular senescence continues to be revealed. m ^6^A can regulate cellular senescence by modulating oxidative stress, telomere length, DNA damage, and senescence-associated secretory phenotypes (SASPs) [9]. However, m ^6^A-mediated senescence of β-cells and endothelial cells promotes the development of diabetes. Furthermore, the m ^6^A-regulated senescence of macrophages and vascular smooth muscle cells (VSMCs) plays an important role in the formation of AS; m ^6^A can influence the development of AD by regulating the senescence of astrocytes [ 10, 11] . Additionally, senescence of m ^6^A-regulated β-cells [12] promotes the development of diabetes by reducing β-cell proliferative capacity and decreasing insulin secretion [13]. Senescence of m ^6^A-regulated VSMCs can promote an inflammatory environment by increasing interleukin 6 (IL-6), IL-8, and other inflammatory factors, thereby promoting atherosclerotic plaque formation [ 14, 15] . Therefore, m ^6^A may be a potential target for the treatment of cellular senescence and age-associated diseases.

In this review, we focus on the regulation of cellular senescence by m ^6^A with regard to telomeres, oxidative stress, DNA damage, and SASP. Additionally, the role of m ^6^A in age-associated diseases (diabetes, AS, AD, and hypertension) is emphasized from the perspective of cellular senescence. Finally, we review the potential challenges and prospects of m ^6^A application in senescence regulation that may provide new clues for the treatment of senescence-related diseases.

## Basic Knowledge of m ^6^A Methylation

**Figure 1:**
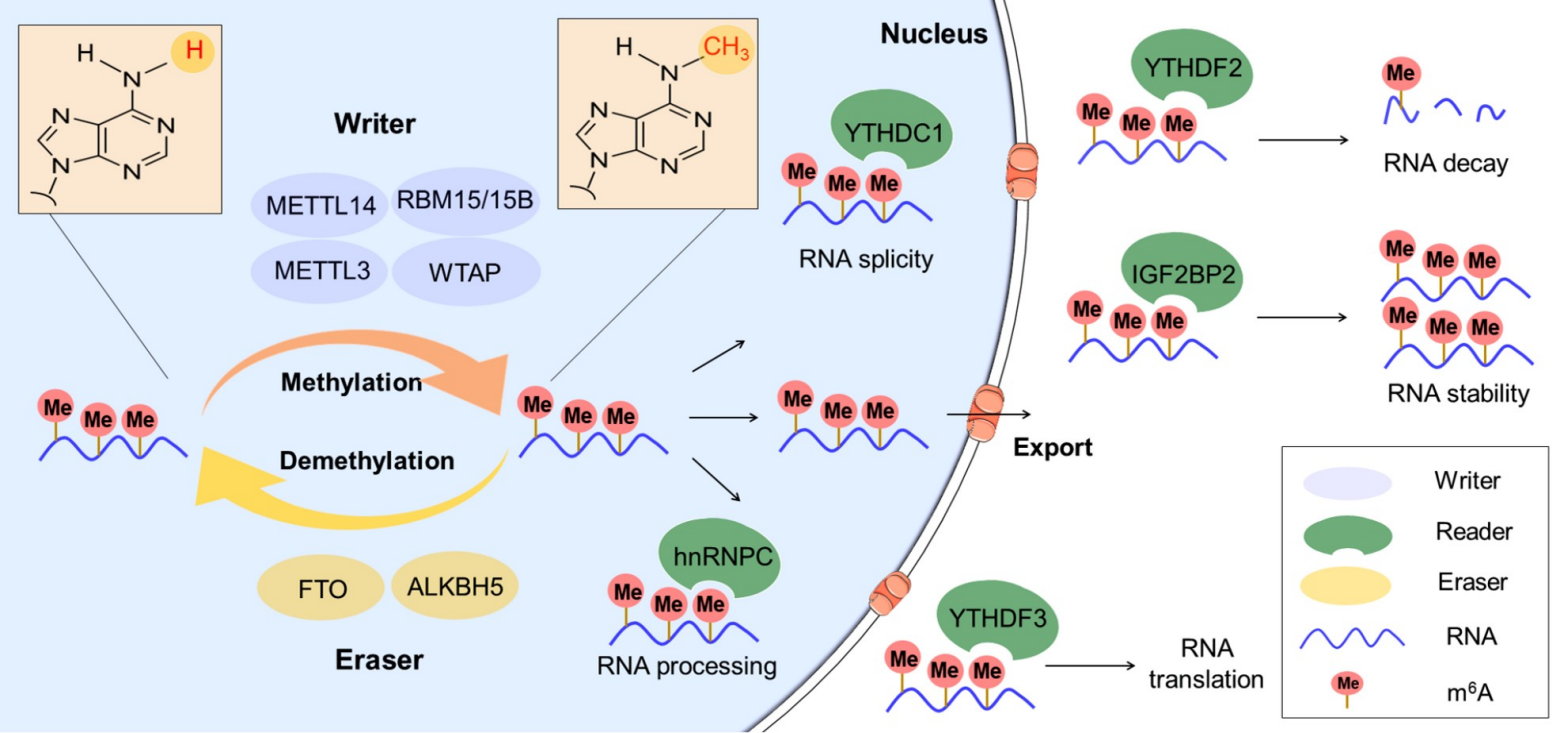
Figure 1 The process of m ^6^A RNA methylation and functions of m ^6^A effector proteins in RNA metabolism The m 6A effector proteins include writers, erasers, and readers. The role of writers, including WTAP, RBM15/15B, METTL3, and METTL14, is to catalyze the m 6A methylation modification of RNA. The role of erasers such as FTO and ALKBH5 is to mediate the demethylation modification of RNA. The role of readers, namely, IGF2BP1/2/3, YTHDC1/2/3, YTHDF1/2/3, HNRNPA2B1, and HNRNPC, is to read the m 6A signal on RNA to regulate RNA splicing, processing, decay, stability and translation, and other biological processes.

Hundreds of chemical modifications have been identified on RNA, including m ^6^A, 5-methylcytosine, N ^1^-methyladenosine, and 5-hydroxymethylcytosine [16]; among these modifications, m ^6^A is the most common and abundant internal transcriptional modification, accounting for approximately 60% of these modifications [ 16– 18] . N6-methyladenosine modification occurs predominantly on adenine in the RRAC sequence and can regulate almost every aspect of mRNA, circRNA, tRNA, and rRNA [ 19, 20] , specifically regulating expression, processing, translation, and decay across these RNA types [ 20– 22] . The corresponding m ^6^A functionality is typically associated with writers, erasers, readers, and related enzymes [23]. The primary role of methyltransferase (writer) is to catalyze m ^6^A methylation modification of RNA *in vivo* and *in vitro* with its major components, WTAP, RBM15/15B, KIAA1429, METTL3, and METTL14 ( Figure 1) [19]. The most studied methyltransferase component is METTL3, which mainly plays a catalytic role by forming complexes with METTL14 [ 19, 20, 24] . Alternatively, demethylases (erasers) mediate demethylation modifications on RNA primarily using the enzymes FTO and ALKBH5 in this process [8]. The function of the reader (m ^6^A recognition protein) is to regulate cellular biological processes by reading m ^6^A signals on RNA [16]. The components of the reader include IGF2BP1/2/3, YTHDF1/2/3, HNRNPA2B1, and HNRNPC [ 19, 25] .

Studies have found that m ^6^A contributes to a variety of biological processes and plays different roles across these different biological processes. For example, the role of m ^6^A in development has been demonstrated in various organisms, such as humans [26], mice [27], goats [28], pigs [29], and bees [30]. Specifically, the importance of m ^6^A in the development of different organs, such as the heart [31], gonads [32], testes [33], brain [34], and cerebellum [35], is gradually being revealed. Moreover, m ^6^A plays an important role in the metabolism of organisms, predominantly in lipid metabolism. In this metabolic process, m ^6^A can regulate the mRNA of genes related to lipid metabolism to regulate lipid production, storage, preadipocyte differentiation, and cholesterol efflux [36]. Additionally, m ^6^A plays an important role in the immune response; specifically, m ^6^A can regulate immune cells, such as macrophages, dendritic cells, lymphocytes and natural killer cells, and participate in the regulation of immunosuppressive molecules, such as programmed death ligand 1 (PD-L1), or immune signalling pathways, such as HIPPO/YAP and WNT/β catenin [37]. Therefore, targeting m ^6^A modifications may be crucial for growth, development, and disease progression.

## Regulatory Role of m ^6^A in Cellular Senescence

**Figure 2:**
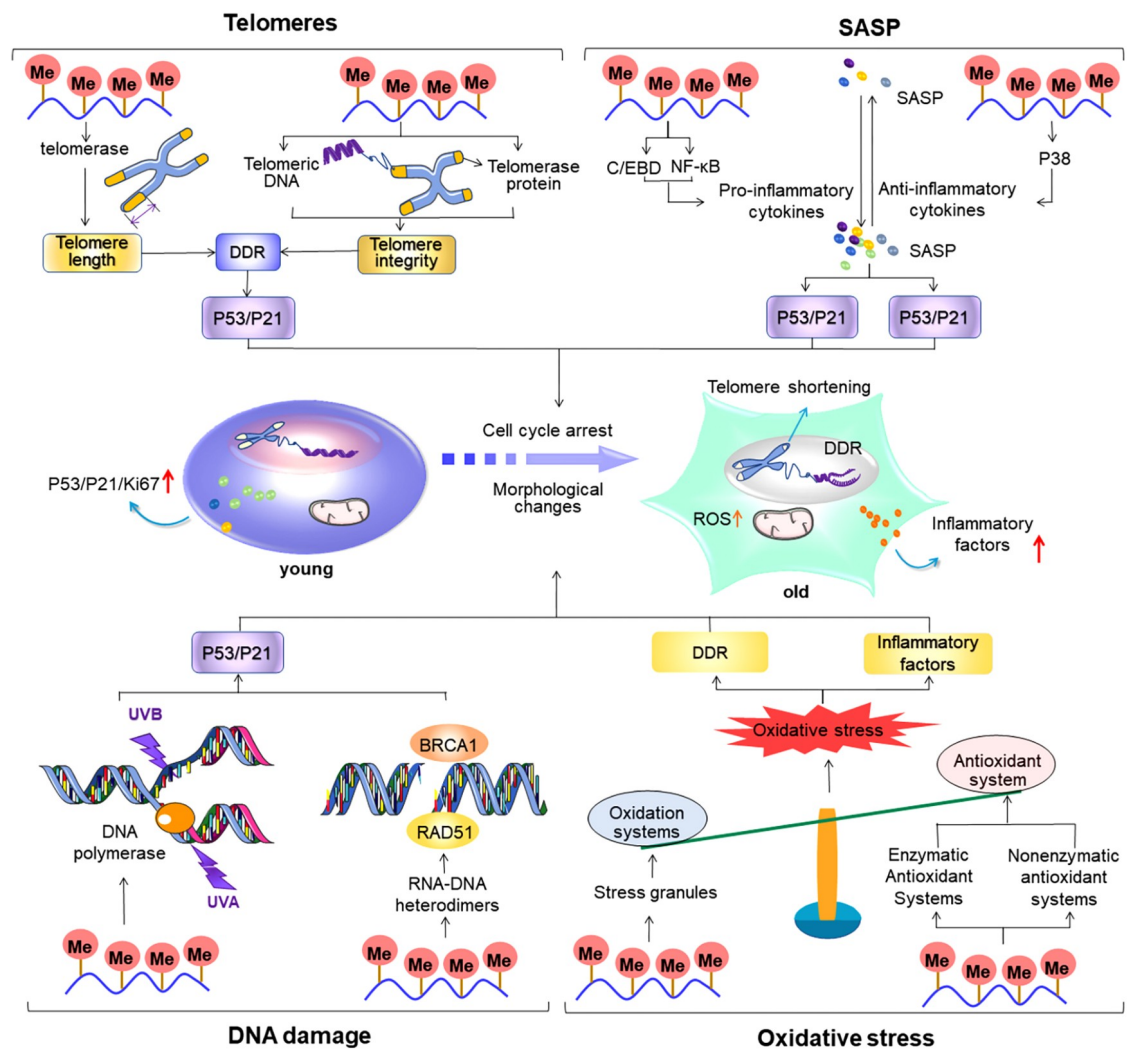
Figure 2 Regulatory role of m ^6^A (N ^6^-methyladenosine) in cellular senescence-related processes Telomere shortening, increased SASP secretion, DNA damage, and oxidative stress are important factors contributing to cellular senescence.Overall, m 6A plays an important regulatory role in these senescence-related processes. During telomere shortening, alterations in m 6A levels regulate the cell cycle; ultimately, this regulates cellular senescence by affecting telomere length and integrity, thereby causing DNA damage and promoting p53/p21 expression. During SASP secretion, m 6A affects the degree of inflammation primarily by regulating the expressions of pro- and anti-inflammatory cytokines; these cytokines, in turn, affect the progression of cellular senescence by regulating p53/p21 and p16 expression to influence the cell cycle. During DNA damage, m 6A regulates p53/p21 expression by influencing the recruitment of DNA polymerase at the site of damage and regulating DNA break repair, thereby regulating cellular senescence. During oxidative stress, m 6A predominantly acts on oxidative and antioxidant systems to regulate the balance of oxidation and antioxidation in vivo; overall, this further influences the degree of DNA damage and inflammation levels and ultimately regulates the process of cellular senescence via cell cycle regulation.

Dysregulation at the transcriptional level and alterations in the translational machinery are key factors contributing to cellular senescence [ 38, 39] , which is strictly controlled by a program that produces the corresponding senescence phenotype. Alterations in some chemical modifications of mRNAs during cellular division can regulate the expression programs of these genes at the transcriptional level by affecting mRNA stability, storage, and translation [40]. N6-methyladenosine methylation is an important epigenetic modification in posttranscriptional regulation, ultimately affecting mRNA stability, storage, translation, and decay [40]. Recently, numerous studies have demonstrated that m ^6^A is involved in the regulation of cellular senescence in human umbilical vein endothelial cells, human mesenchymal stem cells (MSCs), β-cells, and other cells [ 12, 41, 42] ( Table 1). Therefore, we focus on the function of m ^6^A in cellular senescence from the perspective of oxidative stress, telomeres, DNA damage, SASP, and other related molecular processes at a posttranscriptional level ( Figure 2).

**Table:** **Table 1** The role of m ^6^A in processes associated with cellular senescence

Cellular senescence pathways	m ^6^A-related molecules	Expression	m ^6^A level	Main functions	Cell type	Ref.
Oxidative stress	YTHDF	Elevated	Unchanged	Promote the formation of stress particles	Human osteosarcoma cells	[43]
FTO	Elevated	Reduced	Increase expression of superoxide dismutase, catalase, and quinone oxidoreductase	Bovine granulosa cells	[44]
METTL3/14 FTO	Elevated Reduced	Elevated	Promote glutathione expression	Human keratin-forming cells	[45]
DNA damage	METTL3	Elevated	Elevated	Repair DNA damage caused by UV exposure	Human osteosarcoma cells, melanoma cells, hele cells	[46]
ALKBH5	Reduced	Elevated	Protect cells from DNA damage	Human embryonic kidney 293 cells	[47]
METTL3	Elevated	Elevated	Recruit RAD51 and BRCA1 to the DNA break to help with DNA repair	Human osteosarcoma cells	[48]
Telomeres	METTL3	Elevated	Elevated	Inhibit telomere recruitment and regulates telomere length	Human hepatocellular carcinoma cells, human non-small cell lung cancer cells, human prostate cancer cells	[49]
ALKBH5	Elevated	Reduced	Remove m ^6^A from telomerase mRNA to facilitate telomerase breakdown	Human embryonic kidney 293 cells	[50]
METTL3	Elevated	Elevated	Enhance PARP1 stability and maintains telomere integrity	Gastric cancer stem cells	[51]
SASP	METTL3	Elevated	Elevated	Reduce levels of pro-inflammatory cytokines, such as TNF	Macrophages	[ 52, 53]
IGF2BP2	Elevated	Unchanged	Promote IL-17 expression	Mouse embryonic fibroblasts	[54]
METTL3	Elevated	Elevated	Promote the activation of the TRAF6-NF-κB pathway to promote secretion of pro-inflammatory cytokines, such as IL-1β, IL-6, TNF-α and IL-18	Microglia	[55]

### m ^6^A in oxidative stress

Oxidative stress is an imbalance between oxidation and antioxidant action in the body, whereby there is a shift towards increased oxidation [56]. Oxidative stress causes damage to biological systems due to the excessive production of reactive oxygen species (ROS) and reactive nitrogen species (RNS); ultimately, this results in the oxidation of biomolecules such as lipids, proteins, and DNA [ 57, 58] . Oxidative stress can promote cellular senescence by accelerating telomeric wear, promoting DNA damage, and increasing SASP [ 59, 60] . However, the presence of antioxidant systems in the organism can deter the emergence of oxidative stress. Antioxidant systems are divided into the following two main categories: enzymatic and nonenzymatic antioxidant systems. Therefore, regulating the degree of oxidative stress by modulating the balance between oxidative and antioxidant systems is an important way to regulate cellular senescence.

Based on evidence, m ^6^A is altered during oxidative stress. For example, in models of oxidative stress induced using cobalt and nitrite, there were significant differences in m ^6^A levels compared to the normal group; this suggested that these changes may be associated with demethylases (FTO) and methyltransferases (primarily WTAP and METTL14) [ 61, 62] . Additionally, abnormal m ^6^A levels were found in oxidative stress-related aspects of disease lesion processes, such as liver fibrosis and cancer. Moreover, changes in m ^6^A levels may aggravate oxidative stress [ 63– 67] . For example, differential m ^6^A methylation during liver fibrosis is predominantly enriched during oxidative stress [63]. Sun *et al*. [64] further demonstrated that m ^6^A mediated by the presence of YTHDF3 can inhibit oxidative stress by increasing peroxidase expression and reducing ROS accumulation, thereby promoting hepatic stellate cell activation and reducing liver fibrosis. Therefore, targeting m ^6^A to regulate oxidative stress levels may have potential implications for the prevention and treatment of related diseases.

It has been established that m ^6^A plays an important role in both the antioxidant and oxidative systems. In the antioxidant system, m ^6^A can regulate oxidative stress levels via the regulation of related antioxidant genes or enzymatic/nonenzymatic antioxidant systems [ 44, 45] . For example, FTO can enhance the antioxidant system by targeting and regulating m ^6^A to promote the expression of antioxidant enzymes such as superoxide dismutase, catalase, and quinone oxidoreductase 1 [44]. In the oxidative system, m ^6^A regulates oxidative stress levels mainly through modulation of oxidative stress granules. For example, in a study conducted by Fu *et al*. [43], it was demonstrated that m ^6^A, in the presence of YTHDF, promotes the formation of stress granules, which ultimately leads to increased oxidative stress.

Since the level of oxidative stress determines, to some extent, the degree of cellular senescence [ 68, 69] , targeted modulation of oxidative stress levels is one of the potential ways to treat cellular senescence and related diseases; however, several challenges remain. For example, how to accurately locate the site of oxidative stress, determine the level of oxidative stress, and determine the extent of its effect on cellular senescence are current difficulties that remain to be elucidated. Furthermore, since almost all biologically relevant molecules can react with most active free radicals at similar rates, the use of oxidants to scavenge oxygen-containing free radicals, such as ROS, *in vivo* has a limited role in the antioxidant system [70]. However, it is surprising that many studies have revealed mechanisms by which m ^6^A regulates the oxidative and antioxidant systems in multiple ways; overall, this understanding enriches the potential strategies for regulating oxidative stress. With the advancement of targeted drug delivery techniques and the discovery of m ^6^A modulators and related inhibitors, it is expected that targeting m ^6^A to strengthen the antioxidant system and weaken the oxidative system in the same cell could enable efficient regulation of oxidative stress levels. Chen *et al*. [45] demonstrated that m ^6^A can inhibit cell viability by enhancing oxidative stress; however, further studies are required to fully demonstrate that m ^6^A can regulate other key indicators of cellular senescence ( *e.g.*, cell cycle) by modulating oxidative stress levels.

### m ^6^A in DNA damage

DNA damage refers to structural changes in DNA due to specific factors. DNA damage can include mutations, deletions, insertions, inversions or translocations, and double-strand breaks. There are several factors that can cause DNA damage, such as physical and chemical factors; the primary physical factors are UV and ionizing radiation, whereas the primary chemical factors are alkylating agents or synthetic chemicals. In addition, DNA can be damaged spontaneously, for example, by errors in the replication process or by spontaneous chemical changes in the DNA. The rich genetic information of an organism is contained in a specific sequence of DNA bases and DNA structure. DNA is inherently self-repairing when it is affected by external factors; however, when damage cannot be repaired, the corresponding genetic information is permanently altered. DNA damage can also lead to cellular senescence by causing mitochondrial dysfunction, autophagy impairment, metabolic disorder, and other processes by impairing transcription, DNA replication, or epigenetic modifications [ 71, 72] . Therefore, studying the way in which DNA damage is regulated is an important approach to determine how to delay cellular senescence.

Currently, DNA damage repair processes primarily include DNA break repair (single-strand break repair, double-strand break repair, DNA damage, and telomeric repair), base DNA damage repair (reversal of DNA damage and base excision repair), and multiple and bulky base damage repair (including nucleotide excision, mismatch, and interstrand crosslink repair) [73]. In recent years, it has been continuously demonstrated that m ^6^A can contribute to a variety of damage repair processes for multiple classes of DNA. For example, when catalysed by the methyltransferase METTL3, m ^6^A can help repair DNA damage caused by UV irradiation by recruiting DNA polymerase to the site of damage [46]. The results from mouse studies indicated that m ^6^A directly regulates the translation of DNA repair-related proteins after recognition by YTHDF1 in a manner that promotes cap-dependent translation [74]. Zhang *et al*. [48] demonstrated that METTL3 at DNA double-strand breaks was activated by phosphorylation and upregulated m ^6^A levels at the site of damage, resulting in an increase in RNA–DNA heterodimers at the site; these heterodimers recruit RAD51 and BRCA1 to the break and aid DNA break repair. Therefore, regulation of DNA damage repair by m ^6^A is expected to be an important pathway for future use in regulating cellular senescence. However, there are still many challenges in developing this DNA damage repair treatment. For example, DNA damage repair occurs predominantly in the nucleus, whereas targeted drug delivery techniques are still at the cellular level, which makes it difficult to effectively target and regulate specific microstructures within the cell. Additionally, many pathways lead to cellular senescence. Of these pathways, DNA damage is only one factor leading to cell senescence; therefore, regulation of DNA damage alone may not be an effective strategy in regulating cellular senescence. Most importantly, direct evidence that m ^6^A regulates cellular senescence through DNA damage pathways has not been directly demonstrated in vitro or in vivo. Therefore, further investigation is required to confirm this scientific conjecture.

### m ^6^A in telomeres

Telomeres are special ‘caps’ located at the ends of chromosomes that consist of telomeric DNA and shelterin protein complexes. The main function of telomeres is to maintain chromosome integrity and prevent chromosome ends from joining [75]. Telomeric function depends primarily on telomere length and integrity [76]. Telomere length shortens with increasing cell division and decreasing telomerase activity. The integrity of telomeres is also damaged over time. The increase in external stimuli can lead to structural damage and a decrease in the synthesis rate of telomere components, which leads to damage to telomeric integrity. This telomeric shortening and loss of telomere integrity allow DNA double-strand break production, triggering a DNA damage response (DDR) that leads to cell cycle arrest and replicative cellular senescence [ 7, 76] . With the advancement of epigenomic studies, the regulatory role of m ^6^A in telomeres has been gradually revealed [49]. Exploring the specific regulatory mechanisms between m ^6^A, telomeres, and cellular senescence is of great importance for the treatment of cellular senescence and related diseases. In this section, the effects of m ^6^A on cellular senescence through the regulation of telomere length and integrity are specifically addressed.

Telomere length is a key determinant of cellular senescence. The main factors affecting telomere length include telomerase, the number of cell divisions, oxidative stress, and DNA damage. Telomerase, the only intracellular enzyme that can lengthen telomeres, plays a crucial role in the maintenance of telomere length [77]. Current studies have shown that m ^6^A can regulate telomere length by regulating telomerase activity, recruitment, and components; this regulation by m ^6^A is dependent on the expression of enzymes such as METTL3, WTAP, YTHDF2, and ALKBH5 [ 49, 78, 50] . In human hepatocellular carcinoma cells, METTL3 can inhibit telomerase activity by suppressing the expression of the telomerase-related gene CBF5 [78]. Lee *et al*. [49] showed that METTL3 can increase the mRNA degradation of the DNA-binding protein HMBOX1 by upregulating its m ^6^A levels, leading to reduced recruitment of telomeric double-stranded DNA and ultimately preventing telomerase recruitment. Furthermore, this study also demonstrated that reduced telomerase recruitment shortens telomeres, increases p53 expression, blocks the cell cycle, and promotes cellular senescence. Additionally, in combination with regulating oxidative stress and DNA damage, m ^6^A was found to play a regulatory role in other major factors affecting telomere length. With the maturation of telomere length determination methods, such as quantitative polymerase chain reaction (qPCR), terminal restriction fragment (TRF) analysis, single telomere length analysis (STELA), and telomere shortest length assay (TeSLA), it is beneficial to pinpoint telomeres that are at abnormal lengths [79]. In the future, it is expected that the regulation of telomeres by m ^6^A will be used to delay cellular senescence in the clinic; however, various challenges remain. For example, there are many factors that influence telomerase recruitment and activity; therefore, aberrant expression of any one of these factors could influence these processes. Nonetheless, it is unclear whether components other than HMBOX1 and cbf5 are regulated by m ^6^A. Therefore, more evidence is needed to demonstrate that m ^6^A regulates cellular senescence through the regulation of telomere length.

Telomere integrity refers to the structural and functional integrity of telomeric proteins and DNA, which serve to protect chromosomes from loss of genetic material [80]. When telomere integrity is compromised, it accelerates aging by targeting chromosome degradation and telomere shortening [ 81, 82] . With the development of epigenomic techniques and in-depth studies on telomere structure and function, m ^6^A was found to maintain telomere integrity by regulating telomeric DNA cofactors and telomerase under the regulation of METTL3 and YTHDF1. For example, m ^6^A targets the mRNA of the cofactor PARP1 (one of the important DNA breakage molecule receptors of the DDR pathway) under the modification of YTHDF1, thereby mediating DNA damage repair by enhancing the stability of PARP1 [51] and ultimately maintaining telomere stability [83]. Although m ^6^A has not been found to regulate telomere integrity through the regulation of telomeric proteins, several studies have indicated that m ^6^A can regulate telomeric proteins. For example, telomeric proteins are predominantly composed of the shelterin protein complex, consisting of the six subunit proteins TRF1, TRF2, RAP1, TIN2, TPP1, and POT1. Mao *et al*. [84] utilized whole transcriptome analysis and determined that differential expression of m ^6^A may be associated with the RAP1 signalling pathway. Therefore, in the future, it is expected that a combined transcriptomic and telomere length assay technique could be used to accurately identify telomeres in senescent cells that are in abnormal condition; maintenance of telomere integrity using the METTL3-METTL14 complex inhibitor S-adenosylhomocysteine [ 85, 86] and the FTO inhibitor IOX3 [87] could then be used to target and regulate m ^6^A levels, thereby delaying cellular senescence. However, further research is required to resolve current difficulties in this potential treatment. All six subunit proteins of the shelterin protein complex can be key factors affecting telomere integrity, but only one subunit protein, RAP1, has been found to be regulated by m ^6^A; this current understanding can be attributed to the lack of research to confirm the regulatory role of m ^6^A in the other five subunit proteins, TRF1, TRF2, TIN2, TPP1, and POT1. However, there are various epigenetic modifications involved in cellular senescence; in addition to m ^6^A, they include histone modifications and DNA methylation. Therefore, the efficacy of modulating m ^6^A alone to delay cellular senescence is limited; additionally, the use of a specific class of drugs tends to make the organism resistant to that specific drug class. However, the sophistication of transcriptomics and proteomics technologies has made it simple to explore the regulatory role of m ^6^A in proteins such as TRF1 and TRF2. In addition, combined epigenetic therapies have been clinically shown to significantly improve efficacy [ 88, 89] and have demonstrated complementary and synergistic effects on different targets [ 90, 91] .

### m ^6^A in the SASP

The SASP is one of the main features of senescent cells; specifically, the SASP typically refers to cytokines that are secreted by cells during senescence that alter the microenvironment [92]. The biological function of SASP can be contradictory, and SASP is beneficial to humans in promoting wound healing and tissue repair; however, it can alternatively promote chronic inflammation, alter the tissue microenvironment, promote senescent cell accumulation, and cause senescence-associated diseases [ 93, 94] . The cytokines secreted across different ageing processes vary somewhat, and the type of cytokine secreted usually depends on the SASP trigger pathway [95]. DDR, stress kinases, inflammasomes, inflammation, and cell survival-associated transcription factors are common SASP trigger pathways [92]. With increasing age, cellular SASP secretion increases; additionally, increased SASP accelerates senescence of nearby cells through paracrine action [96]. Therefore, it is important to investigate the regulatory mechanisms of SASP to delay cellular senescence. The inflammatory factors that are the main features of SASP are the various cytokines with anti- or proinflammatory effects that participate in and mediate the inflammatory response [97]. The balance of anti- and proinflammatory factors determines the trend of the inflammatory response. Anti-inflammatory factors predominantly include IL-4/10/35 and transforming growth factor β (TGF-β); proinflammatory factors mainly include IL-1β/2/6/15/16/17; tumour necrosis factor (TNF); and interferon γ (IFN-γ) [ 98– 100] . In this section, the production and secretion mechanisms of inflammatory factors in the SASP are discussed, and the epistemic regulatory mechanisms of the SASP are revealed, providing new ideas for the prevention and treatment of aging.

The epigenetic regulation of inflammation has received much attention in recent years, especially with regard to m ^6^A modification [ 101, 102] . For example, in the lipopolysaccharide (LPS)-induced inflammatory response, METTL3 regulates m ^6^A on TNF receptor-associated factor 6 (TRAF6) mRNA; this leads to reduced expression of TRAF6 and inhibition of the NF-κB and MAPK signalling pathways, which ultimately leads to increased inflammation [103]. Recently, m ^6^A was demonstrated to regulate cellular senescence through the regulation of pro- and anti-inflammatory factors. With regard to proinflammatory factors, when under the modification of METTL3 and IGF2BP2, m ^6^A can regulate the secretion of cytokines through the NF-κB and MAPK inflammatory signaling pathways. Specifically, when under METTL3 modification, m ^6^A can regulate the levels of proinflammatory cytokines, such as TNF, by modulating NF-κB and MAPK, thereby regulating LPS-induced macrophage inflammation [ 52, 53] . Increased proinflammatory factors can, in turn, trigger cellular senescence by accelerating telomere shortening, increasing DNA damage, and promoting SASP secretion via the activation of senescence-related pathways such as p16 and p53/p21 [104]. In regard to anti-inflammatory factors, m ^6^A can modulate cytokines through the regulation of the p38 and MAPK inflammatory signalling pathways under METTL3 modification. Elevated METTL3 in human keratinocytes specifically regulates inflammatory responses by upregulating m ^6^A levels to induce increased secretion of the anti-inflammatory cytokine IL-10 [105]. Given that anti-inflammatory cytokines can interact with proinflammatory cytokines to influence the SASP-induced inflammatory response and cellular senescence [ 106, 107] , there is some evidence of a potential role for m ^6^A in cellular senescence by targeting the production of pro- and anti-inflammatory factors. At present, m ^6^A is only superficially studied in other SASPs ( *e.g.*, growth factors, chemokines, and matrix remodelling enzymes); therefore, further confirmation of its role in these SASP factors is needed. As m ^6^A can regulate multiple aspects of RNA metabolism, it is expected that m ^6^A could be effective in delaying cellular senescence by simultaneously regulating multiple SASPs, such as proinflammatory factors, anti-inflammatory factors, growth factors, and chemokines.

## m ^6^A Methylation in Age-related Disease

Cells serve as the basic units of structure and physiology that constitute an organism. An increase in their level of senescence leads to a deterioration in the function of the corresponding organs and tissues and is implicated in AS [ 108, 109] , AD [ 110, 111] , diabetes [ 112, 113] , Parkinson’s disease (PD) [114], chronic obstructive lung disease [115], insulin resistance [116], cancer [ 117, 118] , and osteoporosis [119]. The involvement of mRNA methylation in multiple aspects of cellular senescence provides the basis for a more comprehensive and in-depth exploration of the epigenetic mechanisms underlying various age-associated diseases. Therefore, this section describes the changes in m ^6^A in senescence-associated diseases and addresses the potential mechanisms by which m ^6^A regulates these diseases via the regulation of cellular senescence ( Table 2).

**Table:** **Table 2** The role of m ^6^A in the regulation of age-associated diseases

Diseases	Senescent cells	m ^6^A-related molecules	Expression	m ^6^A level	Effects of m ^6^A on disease through cellular senescence	Ref.
Diabetes	β-cells	METTL3/14	Reduced	Reduced	Regulation of the insulin/IGF1-AKT-PDX1 signalling axis blocks the β-cell cycle and inhibits insulin secretion	[12]
Vascular endothelial cells	METTL3	Reduced	Reduced	Inhibits endothelial cell proliferation, reduces cell viability and migration, leads to vascular endothelial cell senescence, and causes insulin resistance	[120] [121]
AS	VSMCs	YTHDC	Reduced	Reduced	Inhibits smooth muscle proliferation and G1-S phase, leading to vascular smooth muscle cell senescence and promotion of AS development	[122] [123]
Endothelial cells	METTL3	Reduced	Reduced	Promotes the formation of atherosclerotic plaques	[124]
AD	Astrocytes	METTL14	Reduced	Reduced	Prolongs the cell cycle of radial glial cells, causes astrocyte senescence, and promotes the AD development	[125]

### m ^6^A methylation in diabetes

Diabetes mellitus is a metabolic disease that can be caused by the pancreas not being able to produce enough insulin or the body not being able to use this insulin effectively and is, therefore, characterized by higher-than-normal blood glucose ranges. Ageing is considered to be a major risk factor for the development of type 2 diabetes [ 126, 127] . A study of US adults indicated a diabetes prevalence of 2.9% for those aged 20‒44 years, 12.4% for those aged 45‒64 years, and 19.8% for those aged 65 years and older [128]. Cellular senescence is a fundamental mechanism of aging, which has been confirmed to be crucial for the development of diabetes. Specifically, cellular senescence in diabetes-related cells, such as β-cells [129] and endothelial cells [120], accelerates the development of diabetes with increased oxidative stress, DNA damage, and SASP [130]. In recent years, studies have continued to identify abnormal expression of m ^6^A in patients with type 2 diabetes mellitus [131], a mouse model of high-fat diet-induced hepatogenic diabetes [132], and a mouse model of diabetic nephropathy [133]; these changes in m ^6^A expression levels have been determined to further affect hepatogenic diabetes in mouse models [132]. Combined with the important role of m ^6^A in cellular senescence and senescence-related processes (oxidative stress, SASP, and DNA damage) [ 130, 134, 135] , the mechanism by which m ^6^A regulates diabetes through this regulation of cellular senescence is addressed in this section, thereby providing information that can be useful in the development of prevention and treatment strategies for diabetes.

#### m ^6^A regulates diabetes through modulation of β-cell senescence

The primary biological function of β-cells is to synthesize insulin and sense the need for insulin secretion. Since insulin secreted by β-cells is the only hypoglycemic hormone in the body, β-cells plays a crucial role in the regulation of blood glucose in the body [136]. Studies have demonstrated that in β-cells, oxidative stress, DNA damage, and increased secretion of SASP in senescence lead to a reduced proliferative capacity, disturbed transcription and protein homeostasis, and increased β-cell dysfunction; ultimately, this leads to reduced insulin secretion and increased insulin resistance, thereby promoting the development of diabetes [ 13, 129, 137– 139] . In almost all types of diabetes, senescent β-cell numbers are increased, and β-cell function is impaired [140]. Currently, the main strategies for improving β-cell function include culturing pancreatic stem cells for *in vitro* regeneration and using the patient’s own stem cells to induce differentiation of these stem cells into β-cells and induce β-cell proliferation *in vivo*. However, each of these approaches has its own limitations. For example, the use of stem cells for *in vitro* regeneration therapy may be associated with artificially uncontrollable proliferation and differentiation of stem cells, alongside the application of other stem cells, which may be highly tumorigenic. Removal of senescent β-cells has been shown to be beneficial in improving β-cell function and preventing the development of diabetes [13]. Therefore, modulation of β-cell senescence is a key strategy to treat diabetes.

With an increasing focus on epigenomic studies, m ^6^A has been found to regulate β-cells in both physiological and pathological states. The levels of m ^6^A, METTL3, METTL14, ALKBH5, and YTHDF1 have been observed to be lower in the β-cells of patients with diabetes than in healthy participants; in contrast, there is no observable change in α-cells between these two groups [12]. Recently, m ^6^A has been suggested to regulate β-cell senescence by regulating the cell cycle and proliferation of these cells. For example, Jesus *et al*. [12] demonstrated that by targeting METTL3 and METTL14, the insulin/IGF1-AKT-PDX1 signalling axis is regulated, resulting in the downregulation of m ^6^A levels and impairment of cell cycle arrest and glucose-stimulated insulin secretion in β-cells. Furthermore, m ^6^A modification by the reader IGF2BP2 can regulate β-cell proliferation by regulating PDX1 [141]. Although there are no current studies that directly demonstrate that m ^6^A can regulate diabetes through the regulation of β-cell senescence, the differential expression of m ^6^A upon regulating key indicators of cellular senescence and diabetic β-cells demonstrates the potential value of m ^6^A in this process.

#### m ^6^A regulates diabetes by modulating endothelial cell senescence

Endothelial cells are a layer of cells in the intima of blood vessels that reduce vascular permeability, are antithrombotic, regulate VSMCs and are involved in the regulation of signalling, immunity, and inflammation [142]. When endothelial cells are dysfunctional, they may cause insulin resistance via increased levels of inflammation and oxidative stress and reduced endothelial-mediated vasodilation; ultimately, this can lead to the development of diabetes [143]. Furthermore, it has been found that endothelial dysfunction usually precedes the onset of diabetes [120]. Endothelial senescence, caused by increased levels of oxidative stress with aging and telomere shortening, is one of the key factors contributing to endothelial cell dysfunction and diabetes. Therefore, exploring the regulatory mechanisms of endothelial cell senescence is beneficial for the prevention and treatment of diabetes.

m ^6^A has been observed to be involved in the regulation of endothelial cell senescence. For example, Li *et al*. [144] found that m ^6^A can delay endothelial cell senescence by regulating the expression of p21 and p16, whereas FTO can promote endothelial cell senescence. In endothelial cells, METTL3-, METTL14-, YTHDF2-, and IGF2BP2-mediated m ^6^A modifications are involved in the regulation of cellular senescence–associated pathways, such as proliferation, migration, inflammation, and viability; additionally, corresponding studies have indicated that m ^6^A can regulate endothelial cell senescence [ 41, 133, 121] . For example, m ^6^A levels within retinal microvascular endothelial cells (RMEC) were observed to be higher in a mouse model of diabetes than in normal controls, suggesting that the targeting of m ^6^A on integrin ( *ITGB1*) mRNA by YTHDF2 resulted in inhibition of RMEC proliferation and migration [145]. This study suggests that m ^6^A may influence the development of diabetes and its complications by regulating RMEC senescence. Fan *et al*. [38] showed that dasatinib and quercetin alleviate HUVEC senescence in a YTHDF2-dependent manner through the TRAF6-MAPK-NF-κB axis of the inflammatory signalling pathway. Furthermore, Yao *et al*. [121] demonstrated that reduced expression of the m ^6^A methyltransferase METTL3 inhibited endothelial cell viability, proliferation, and migration *in vitro*. Although these studies demonstrated to some extent that m ^6^A can regulate key indicators of cellular senescence, they failed to demonstrate significant changes in the endothelial cell senescence cycle and expression of p53, p21, p16 and other key senescence indicators following this m ^6^A regulation of inflammation, proliferation, and migration in these same cells. Therefore, future studies are needed to further demonstrate that m ^6^A regulates the proliferation and migration of RMEC and other key senescence indicators and thus clearly reveal whether m ^6^A can regulate endothelial cell senescence and diabetes.

### m ^6^A methylation in AS

AS is a chronic inflammatory disease that refers to atherosclerotic lesions within the vessel wall due to plaque accumulation [146]. AS formation is initiated by smooth muscle cell and macrophage dysfunction or senescence; specifically, this is caused by elevated levels of inflammation and oxidative stress and dysregulated lipid metabolism [ 147, 148] . Cellular senescence can drive AS in human premature aging syndromes, characterized by a marked accumulation of early senescent cells that lead to an increased incidence of this disease [ 149, 150] . Notably, modulating or targeting the removal of senescent cells has been identified as a potential therapy for AS [151], and progress has been made in the development of drugs with anti-ageing properties, such as polyphenols, metformin, and rapamycin [ 152, 153] . Alternative antiaging drugs, such as quercetin, laccasein, and curcumin, have also been proposed for the treatment of AS [154]. The limitations of drug side effects and targeted delivery techniques have led to these current approaches being ineffective in clearing senescent cells and treating AS. With advances in epigenomic technologies, an increasing number of studies have identified a regulatory role for m ^6^A in cellular senescence and AS. For example, m ^6^A and proteins such as WTAP, METTL3, METTL5, and YTHDF2 are significantly differentially expressed in healthy individuals when compared to corresponding expression levels in patients with AS [155]. Targeted regulation of m ^6^A and its related proteins ( *e*. *g*., METTL3 and METTL14) is effective in improving AS and its associated cardiovascular diseases. For example, increased m ^6^A modification of *FOXO1* mRNA promotes the formation of AS plaques [156]. Alternatively, *METTL3* knockdown has been observed to block AS progression by inhibiting the JAK2/STAT3 pathway via IGF2BP1 [108]. Therefore, this section summarizes the specific mechanisms by which m ^6^A regulates AS through modulation of cellular senescence.

VSMCs are one of the components of the vascular mesothelium and play an important role in a variety of physiological processes. VSMCs are a major cell type involved in the atherosclerotic process; these cells are important throughout the entire development of AS and undergo phenotypic transformations in AS plaques [122]. VSMC senescence in human AS is widely accepted. Matthews *et al*. [157] identified a significantly increased number of senescent VSMCs in advanced human AS lesions compared to that in healthy vessels and showed replicative VSMC senescence in human AS. Furthermore, Grootaert *et al*. [15] established that there was a significantly increased number of senescent VSMCs in AS plaques in a mouse model of AS compared to normal mice. VSMC senescence can promote AS through lipid-mediated oxidative DNA damage and telomere dysfunction [15]. Therefore, delaying VSMC senescence may be an important way to treat AS. For example, in a study conducted by Grootaert *et al*, the deacetylase SIRT6 was shown to protect smooth muscle cells from senescence and reduce AS [15]. However, current *SIRT6* have limited effects in regulating VSMC senescence and AS. Therefore, it is important to explore the mechanisms that effectively delay VSMC senescence for the treatment of AS.

As the physiopathological function of m ^6^A has been studied extensively, the function of m ^6^A in regulating VSMC senescence and AS has been confirmed. The regulation of AS by m ^6^A is dependent on METTL3, METTL14, and IGF2BP1. For example, under METTL3 modification, m ^6^A can stabilize atherosclerotic plaques by regulating the miR-375-3p/PDK1 axis [158]. VSMC senescence by m ^6^A is predominantly regulated by the modification of METTL3, METTL14, WTAP, and FTO, which regulate the VSMC longevity gene *SIRT6*, proliferation, migration, and inflammation [ 11, 14, 15, 159] . Promotion of WTAP inhibits the viability, proliferation, and migratory potential of VSMCs; in contrast, inhibition of WTAP restores the total panaxoside (TPNS)-induced inhibition of cell viability, proliferation, and migratory potential of VSMCs [160]. *METTL3* knockdown has been observed to inhibit the proliferation and migration of human coronary artery smooth muscle cells (HCASMCs) through downregulation of m ^6^A level and has been found to play a role in AS [14]. Nonetheless, this study did not directly demonstrate that m ^6^A can regulate senescence in HCASMCs due to the reduced proliferation and migration capacity that is characteristic of senescent cells; however, it could also go some way to suggest that m ^6^A regulates senescence in HCASMCs. Alternatively, Du *et al*. [161] determined that METTL14 regulates SIRT6 in hepatoma cells by regulating m ^6^A levels on *USP48* (ubiquitin-specific peptidase 48) mRNA; in another study, it was shown that SIRT6 protects smooth muscle cells from senescence and reduces AS. Therefore, it is hypothesized that a METTL14-m ^6^A-SIRT6-VSMC cellular senescence mechanism may exist in VSMCs. Nonetheless, further demonstration of the differential expression of m ^6^A in senescent VSMCs with AS plaques is needed to more wholly determine whether m ^6^A can regulate AS through modulation of VSMC senescence and to demonstrate that VSMC senescence markers and AS markers are altered accordingly in correspondence with m ^6^A levels.

### m ^6^A methylation in AD

AD is a neurodegenerative disease associated with human aging and is an important contributor to dementia [ 162, 163] . Factors currently considered to contribute to AD include cellular senescence, mitochondrial dysfunction, DNA damage, cholinergic dysfunction, inflammation, tau protein phosphorylation, β-amyloid (Aβ) aggregation, and neurotoxicity [ 164– 167] . Because of the complex pathogenesis of AD, corresponding treatment is currently limited to only two classes of cholinesterase inhibitors, such as donepezil, and N-methyl-D-aspartate receptor antagonists, such as memantine [ 168, 169] . Therefore, it is of great importance to clarify the exact mechanism of AD pathogenesis and to develop novel drugs for its treatment. As AD research progresses, the senescence of cells, such as astrocytes, microglia, and neural stem cells, has been implicated in the development of AD; further studies have demonstrated that cellular senescence promotes AD development by promoting Aβ and tau protein lesions [170]. Moreover, removal of senescent cells has been shown to cause a resulting reduction in Aβ and tau protein lesions in the brain; overall, this resulted in improved memory in mouse models of AD [ 171– 173] . Recently, it was found that m ^6^A plays an important role in cellular senescence and AD [174]. Therefore, this section integrates the regulatory relationship between m ^6^A, cellular senescence, and AD to provide an important reference for AD treatment.

Astrocytes are abundant neuroglia in the central nervous system [175], with key roles in maintaining neuronal viability and transmitter metabolism and participating in signalling [ 176, 177] . When astrocytes age, they contribute to the development of AD through increased SASP, Aβ accumulation, tau protein phosphorylation, synaptic dysfunction, neuronal loss, and deposition of neurogenic fibrillary tangles [ 178, 179] . The number of senescent astrocytes in brain tissue increases with age, and the number of senescent astrocytes in brain tissue is significantly higher in patients with AD than in patients without AD of the same age [179]. Bussian *et al*. [171] determined that clearing senescent astrocytes in a mouse model of tau-dependent neurodegenerative disease facilitated improvements in cognitive function in patients with neurodegenerative diseases, such as AD. Therefore, targeted modulation of astrocyte senescence is a potential strategy for AD treatment.

The regulatory role of m ^6^A in astrocytes and AD has been gradually revealed. For example, Cockova *et al*. [180] found that the level of FTO was significantly elevated in astrocytes in an AD model; therefore, it was hypothesized that FTO targets m ^6^A to regulate the development of AD. m ^6^A has been determined to have the potential to regulate astrocyte senescence by regulating the astrocyte cell cycle and the corresponding SASP. For example, radial glial cells are a specific type of astrocyte [181]; a corresponding knockdown of *METTL14* decreased m ^6^A level and prolonged the cell cycle in this cell type [125]. Another study noted that during inflammation induced by LPS stimulation of human astrocytes, elevated levels of METTL3 and m ^6^A can suppress the inflammatory response by inhibiting the expression of the inflammatory vesicle NLRP3. Additionally, it was demonstrated that inhibition of the inflammatory response may be associated with reduced levels of proinflammatory factors, such as IL-6 and TNF-α, and increased levels of the anti-inflammatory factor IL-10 [182]. Since elevated m ^6^A levels lead to altered expression levels of SASP factors, such as IL-6 and TNF-α, it is hypothesized that m ^6^A may be involved in the regulation of human astrocyte senescence. In conclusion, it is postulated that m ^6^A may regulate the development of AD by modulating the senescence regulation of glial cells. However, it is still unclear whether m ^6^A plays a regulatory role in oxidative stress, DNA damage, mitochondrial dysfunction, and other key factors that contribute to astrocyte senescence and AD. At present, two key difficulties need to be addressed to allow full demonstration of the potential m ^6^A-dependent mechanisms in astrocyte senescence and AD. First, in astrocytes, the modulation of more typical indicators of senescence by m ^6^A still needs to be demonstrated, such as p53/p21, p16, and LaminB1, or the number of positive SA-β-gal in astrocytes. Second, it is necessary to demonstrate whether alterations in m ^6^A levels in AD models significantly affect the number of senescent glial cells and the development of AD. Notably, advances in proteomics, transcriptomics, and molecular biology techniques have made it easier to address these difficulties.

## Perspectives and Challenges

With the emergence of an increasing aging population, the prevention and treatment of age-associated diseases have become a crucial focus in current research. Many studies have made significant progress in elucidating the role of m ^6^A and cellular senescence in age-associated diseases [ 15, 183] . With advances in epigenomic techniques, the regulatory role of m ^6^A in cellular senescence is gradually being revealed, and the potential mechanisms of m ^6^A-dependent cellular senescence-age-associated diseases are gaining increasing attention [9]. The use of transcriptomic techniques, hydrogen peroxide, oncogene-induced cellular senescence models, and shock stress-induced AS models are expected to be tools for the future treatment of senescence-related diseases [184]. Given that cellular senescence is caused by a combination of alterations, such as increased oxidative stress, increased DNA damage, telomere shortening, and increased SASP, the effect of regulating cellular senescence from a single aspect is limited. Therefore, the biggest challenge in this field is how to effectively delay cellular senescence through simultaneous modulation of multiple factors. As m ^6^A plays a regulatory role in several key factors of cellular senescence, targeting m ^6^A to regulate cellular senescence is a promising strategy in the treatment of age-associated diseases.

More importantly, m ^6^A regulators and their related inhibitors, such as cyclophosphamide [185], the METTL3-METTL14 complex inhibitor S-adenosylhomocysteine [ 186, 187] , and the FTO inhibitor FB23-2 [188], continue to be discovered; these findings continue to reveal new directions for future cellular senescence studies. With advances in targeted drug delivery technologies, such as liposomes, receptor-targeted drug delivery, milliparticulate formulations, and special carriers [189], it is expected that the future regulation of m ^6^A levels in specific organs, tissues, and cells will be developed to reduce cellular senescence across these specific sites. Several m ^6^A-regulated proteins ( *e*. *g*., SIRT1) have been identified to influence the development of senescence-related diseases by regulating cellular senescence [ 190, 191] ; to some extent, this discovery reveals the potential mechanism of m ^6^A-dependent cellular senescence-related diseases. Additionally, this review provides the first summary of m ^6^A-regulated senescent cells in senescence-associated diseases; overall, this is important for future research in understanding potential targets to regulate cellular senescence in the treatment of senescence-associated diseases. However, there are still many questions that need to be addressed to utilize m ^6^A as a targeted treatment for senescence-related diseases. First, the development of senescence-related diseases is typically associated with the senescence of multiple cells; therefore, targeted administration of m ^6^A requires the use of multiple different targeted delivery modalities to effectively regulate these different cell types, which is a considerable challenge for current research. Second, the use of m ^6^A modulators to target specific cells lacks specificity; therefore, this approach may activate nonsense sequences in genes, causing unpredictable consequences. Third, senescent cells may be caused by dysregulation of m ^6^A levels within one or more organelles; because m ^6^A plays multiple roles within a given organelle, targeting and regulating m ^6^A levels in a specific organelle and reducing side effects present complications that need to be addressed. Interestingly, with the continuous development of epigenetic and experimental techniques, there is an increase in studies that use targeted RNA modifications to regulate inflammation and related diseases [ 192, 193] . It is beneficial to use targeted m ^6^A RNA modifications to regulate the secretion of inflammatory factors to reduce cellular senescence; furthermore, targeting m ^6^A RNA modification can help to improve immune function decline and metabolic abnormalities due to alterations in m ^6^A regulators. In addition, with the advancement of combination drug therapies, it is necessary to achieve the simultaneous targeting of multiple senescent cells associated with senescence-related diseases and multiple organelles within these cells to effectively regulate these diseases.

Notably, in addition to the regulatory mechanisms of m ^6^A-β-cell/vascular endothelial cell senescence-diabetes, m ^6^A-vascular smooth muscle/endothelial cell senescence-atherosclerosis, and m ^6^A-astroglial cell senescence-Alzheimer’s disease discussed in this review, the regulatory roles of m ^6^A and cell senescence have been observed in osteoarthritis (OA) [183], intervertebral disc degeneration (IVDD) [ 194– 196] , osteoporosis [197], and other musculoskeletal system degenerative diseases [ 195, 196] . For example, several studies have shown dysregulated expression levels of METTL3, FTO, and YTHDF2 in patients with OA and animal models of OA [ 198– 200] . Another study showed a significant increase in senescent fibroblast-like synoviocytes (FLSs) in patients with OA and mouse models of OA and further clarified that METTL3-mediated m ^6^A regulates autophagy by affecting the stability of ATG7 mRNA, thereby regulating FLS senescence and OA progression by affecting SASP secretion [183]. The prevalence of IVDD typically increases with age, and a number of studies have now confirmed that nucleus pulposus cell (NPC) senescence is an important cause of IVDD [ 201– 203] . Li *et al*. [194] found that m ^6^A levels were dysregulated in senescent NPCs. Animal experiments revealed that targeting m ^6^A on lncRNA NORAD regulates E2F3, a key regulator of the cell cycle via PUM1/PUM2, thereby affecting NPC senescence and ultimately regulating IVDD [194]. Another study that focused on NPC senescence revealed a novel mechanism by which m ^6^A, regulated by ALKBH5, targets NPC senescence via the DNMT3B/E4F1 pathway to regulate IVDD [195]. Bone formation-related cell ( *e.g.*, osteoblasts, bone lining and MSCs) senescence is an important factor that contributes to osteoporosis [204], and it has been demonstrated that senescent cell removal can reduce the development of osteoporosis [205]. In recent years, the regulatory role of m ^6^A in osteoporosis has also been consistently demonstrated [ 119, 206, 207] . In a study by Wu *et al*. [197], animal experiments were conducted and confirmed that Mettl3 deficiency in bone marrow MSCs leads to osteoporosis, demonstrating to some extent the feasibility of targeting m ^6^A to regulate bone formation-related cells for the treatment of osteoporosis. However, in this study, the role of m ^6^A in the regulation of osteoporosis was not demonstrated to be related to the senescence of bone marrow MSCs. Notably, in another study, METTL14 upregulation was noted to promote bone marrow MSC proliferation [208]. m ^6^A may play a regulatory role in bone marrow MSC senescence, and a large amount of research evidence is still required to confirm the therapeutic strategy for m ^6^A-bone marrow MSC osteoporosis. In conclusion, the above studies revealed the potential value of targeting m ^6^A to regulate senescence-related musculoskeletal system degenerative diseases from a cellular senescence perspective.
